# Adapting to CONNECT: modifying a nursing home-based team-building intervention to improve hospital care team interactions, functioning, and implementation readiness

**DOI:** 10.1186/s12913-022-08270-1

**Published:** 2022-07-29

**Authors:** Virginia Wang, Joshua D’Adolf, Kasey Decosimo, Katina Robinson, Ashley Choate, Rebecca Bruening, Nina Sperber, Elizabeth Mahanna, Courtney H. Van Houtven, Kelli D. Allen, Cathleen Colón-Emeric, Teresa M. Damush, Susan N. Hastings

**Affiliations:** 1 Health Services Research and Development Center of Innovation, Durham VA Health Care System, Durham, NC USA; 2 Department of Population Health Sciences, Duke University School of Medicine, Durham, NC USA; 3 Department of Medicine, Duke University School of Medicine, Durham, NC USA; 4 Department of Medicine & Thurston Arthritis Research Center, University of North Carolina at Chapel Hill, Chapel Hill, NC USA; 5 Geriatric Research Education and Clinical Center, Durham VA Health Care System, Durham, NC USA; 6 Health Services Research and Development Center for Health Information and Communication, Richard L. Roudebush VAMC, Indianapolis, IN USA; 7 Department of General Internal Medicine and Geriatrics, Indiana University School of Medicine, Indianapolis, IN USA; 8 Regenstrief Institute, Inc., Indianapolis, IN USA; 9 Center for the Study of Aging and Human Development, Duke University School of Medicine, Durham, NC USA

**Keywords:** Adaptation, Complexity science, Teams, Mobility, Implementation, Intervention design

## Abstract

**Background:**

Clinical interventions often need to be adapted from their original design when they are applied to new settings. There is a growing literature describing frameworks and approaches to deploying and documenting adaptations of evidence-based practices in healthcare. Still, intervention modifications are often limited in detail and justification, which may prevent rigorous evaluation of interventions and intervention adaptation effectiveness in new contexts. We describe our approach in a case study, combining two complementary intervention adaptation frameworks to modify CONNECT for Quality, a provider-facing team building and communication intervention designed to facilitate implementation of a new clinical program.

**Methods:**

This process of intervention adaptation involved the use of the Planned Adaptation Framework and the Framework for Reporting Adaptations and Modifications, for systematically identifying key drivers, core and non-core components of interventions for documenting planned and unplanned changes to intervention design.

**Results:**

The CONNECT intervention’s original context and setting is first described and then compared with its new application. This lays the groundwork for the intentional modifications to intervention design, which are developed before intervention delivery to participating providers. The unpredictable nature of implementation in real-world practice required unplanned adaptations, which were also considered and documented. Attendance and participation rates were examined and qualitative assessment of reported participant experience supported the feasibility and acceptability of adaptations of the original CONNECT intervention in a new clinical context.

**Conclusion:**

This approach may serve as a useful guide for intervention implementation efforts applied in diverse clinical contexts and subsequent evaluations of intervention effectiveness.

**Trial registration:**

The study was registered at ClinicalTrials.gov (NCT03300336) on September 28, 2017.

## Background

Clinical interventions need to be adapted from their original design when they are applied to new settings. Modifications that consider new clinical and local environmental contexts are critical to successful implementation, while at the same time maintaining fidelity to core components of the practice or intervention’s original design [1–3]. In order to support evaluations of intervention effectiveness and to inform future expanded implementation efforts [4], documenting changes and rationale to interventions are paramount [5]. In the last decade, the evolving field of implementation science spawned increasing attention to intervention adaptation. There is a growing literature describing frameworks and approaches to deploying and documenting adaptations of evidence-based practices in healthcare [6–10]. Yet, reporting of intervention adaptations (and their justification) is limited in detail and therefore hampers robust evaluation of intervention and adaptation effectiveness in new contexts [11, 12]. This critique suggests gaps in approaches to modifying interventions for new application.

In this paper, we describe a systematic process of intervention adaptation and documentation that is useful for evaluating intervention effectiveness and informing refinements for continued dissemination and implementation efforts. Specifically, we present a case study where we combined two approaches for systematic intervention adaptation and detailed documentation of modifications. Guided by the Planned Adaptation Framework [6] and the expanded Framework for Reporting Adaptations and Modifications [9, 13], we describe our approach to adapting a provider-facing team building and communication intervention for application in a new clinical setting. *CONNECT for Quality* is an intervention designed to serve as a foundation for implementing new practice [14]. Specifically, *CONNECT for Quality* is informed by complexity science and social learning theories [15–17], which describe learning as a social process involving staff interactions, relationship networks, communication and information flow to support uptake of new clinical practice [14, 18]. The *CONNECT for Quality* intervention is designed to promote connection, information flow, and use of cognitive diversity to promote team function and readiness for implementing quality improvement programs in nursing homes [14, 18]. In this way, it is a critical precondition of effective implementation of new practice that is highly relevant for scaling up new programs in other clinical contexts (i.e., addresses the system problems that emerge during full-scale implementation). We describe the scale out of *CONNECT for Quality* for application in a new clinical context of hospital inpatient care teams, employing a systematic process for identifying, justifying, and documenting its modifications. We also report the initial experience of CONNECT delivery at hospitals and describe how this systematic process of intervention adaptation and documentation is useful for evaluating intervention effectiveness and informing refinements for continued dissemination and implementation efforts. This approach serves as a guide for intervention implementation efforts applied in diverse clinical contexts.

### *CONNECT* for *Quality* for nursing home falls prevention

We adapted *CONNECT for Quality*, an intervention originally designed to promote team function and readiness for implementing quality improvement efforts in nursing homes [19]. *CONNECT for Quality* is a bundle of interaction-oriented activities to enhance communication and decision-making among clinical staff. Broadly, the intervention comprises several types of activities:

- Group-based sessions designed with didactic and experiential activities to increase connections and information flow between providers and encourage them to seek out alternative explanations from others to make sense of new clinical data.
- Facilitator-led, group sessions using storytelling and role play to practice new behaviors.
- Individual sessions in which external facilitators assist staff to map their relationships and communication patterns, discuss strategies for creative problem solving around communication barriers, and provide mentorship to sustain new interaction behaviors.

In a randomized study, *CONNECT for Quality* was associated with improvements in communication and decision-making among clinical staff and in patient outcomes in nursing home quality improvement [14, 20, 21]. Accordingly, we consider *CONNECT for Quality* to be an essential process for the creation of functional relationship networks and communication channels for learning, information exchange, and problem solving for rollout of new clinical programs in other clinical and implementation contexts.

### Applying *CONNECT for Quality* to improve inpatient mobility: *CONNECT for STRIDE*

In partnership with the creators of *CONNECT for Quality*, United States Department of Veterans Affairs (VA) national health care system’s national clinical leaders, and local clinical champions, *CONNECT for Quality* was modified for use in a multi-site implementation project to enhance communication and coordination among interdisciplinary care teams implementing STRIDE (assiSTed eaRly mobIlity for hospitalizeD older vEterans), a new clinical program for hospitalized patients. STRIDE is a supervised walking program for hospitalized older adults focused on maintaining musculoskeletal strength and mobility during hospitalization. Originally developed and tested at the Durham VA Healthcare System in 2012, STRIDE consists of a one-time gait and balance assessment conducted by a physical therapist followed by daily supervised walks for the duration of the hospital stay with a mobility assistant (most commonly a therapy or nursing assistant) [22]. Initial results of STRIDE included improved likelihood of discharge to home (vs. skilled nursing or rehabilitation facility) and reduced length-of-stay [22], which led to efforts to expand STRIDE to other VA hospitals. Initial program experience also indicated the importance of inter-professional relationships and team dynamics among the clinical delivery team as a key determinant of success. However, a common barrier to effective implementation of new clinical programs is a focus on the clinical program content while ignoring the organizational learning context and processes needed to successfully implement change.

In anticipation of this challenge, we paired *CONNECT for Quality*, as an adjunctive effort, to facilitate and scale-up the implementation of STRIDE in hospitals. Therefore, *CONNECT for Quality* was adapted into *CONNECT for STRIDE* to serve as a team “booster” in hospital wards to address challenges related to assembling interdisciplinary teams of providers and carrying out new processes for delivering mobility assistance. Hospitals were eligible to participate if they had a minimum average daily census of 20 general medicine patients per day (approximately 250 patients in a 3-month period), agreed to start the STRIDE program during their randomly assigned 3-month implementation and launch time period for STRIDE [19, 23], and were willing to receive and participate in the *CONNECT for STRIDE* intervention. Half of all STRIDE participating hospital sites were randomly assigned to receive *CONNECT for STRIDE*.

The context for delivering *CONNECT for Quality* to the STRIDE program’s clinical staff was a significant departure from its original setting and audience (Table 1). Specifically, *CONNECT for Quality* was developed to augment quality improvement efforts of existing clinical responsibilities (i.e., falls prevention) in nursing homes. In contrast, *CONNECT for STRIDE* is delivered as a primer to a new clinical program and service delivered in VA hospital general medicine inpatient wards that require new clinical processes – including new tasks and roles for some staff at hospitals – delivered to eligible hospitalized patients. Second, *CONNECT for Quality* was designed for a target audience of all clinical and non-clinical staff in nursing homes to address hierarchical management structures and poor connection across silos which negatively impacted the safety climate around falls prevention and care quality. *CONNECT for STRIDE* is delivered to either existing or newly formed teams of clinicians from medicine, nursing, and physical and rehabilitation therapy. For implementing STRIDE, the primary goal of *CONNECT for STRIDE* was to promote clarity of new roles and facilitate communication of relevant clinical information (e.g., patient function and eligibility for in-hospital mobility, prescribed “dose” of STRIDE walking) across practitioners from multiple disciplines involved in inpatient mobility [24].

**Table 1:** Context of clinical settings for CONNECT interventions

Intervention Context	CONNECT for Quality	CONNECT for STRIDE
Clinical Setting	Nursing Home	Hospital Inpatient General Medicine Ward
Clinical Program Tasks	Existing task: Falls prevention	New task: Supervised walking
Team Formation and Processes^a^	Existing team formation/structure New team processes	Existing team formation/structure New team processes
Boundary spanning^b^	No	Yes
Team Membership: Roles in program delivery	• Registered Nurse, Licensed Practice Nurse: fall risk factor assessment and intervention focusing on orthostatics, gait, toileting, medications, environmental hazards • Nursing Assistant: fall risk factor identification and intervention focusing on gait, footwear, toileting, hip protectors, and environmental hazards • MD/NP/PA prescriber and Pharmacist: risk factor assessment, risk factor reduction focusing on psychotropic medication reduction and Vitamin D • Non-clinical Staff (e.g., dining, environmental services, activities): identify environmental hazards, communicate changes in resident status	• Physician (General Medicine): referral • Registered Nurse: coordinate patient/visit schedules • Physical Therapist: initial evaluation, oversight for walking assistant • Licensed Practice Nurse, Certified Nursing Assistant, Physical Therapist Assistant: supervising walks • Unplanned, hospital-led and site-specific additions to STRIDE teams: Speech pathologists Kinesiology technician Safe patient handling and mobility coordinator • Program managers (Registered Nurse, Physical Therapy)
Communication challenges in program delivery	• Insufficient connections (e.g., quality of interactions) between nursing home staff across roles • Communicating relevant clinical information • Limited use of diverse perspectives and interdependent interaction for problem solving	• Clarity of roles • Communicating relevant clinical information and “prescribed dose” of STRIDE

^a^Team formation describe whether new care teams or structures were formed for new clinical program delivery. Team processes describe whether healthcare teams developed new processes (e.g., operational, clinical) for delivering care

^b^Boundary spanning refers to the extent to which tasks and interactions (e.g., care delivery, evaluation, coordination) involve interdependencies with external groups or expertise (e.g., clinical specialty, professional roles) in order to meet clinical program objectives

## Methods

**Fig. 1:**
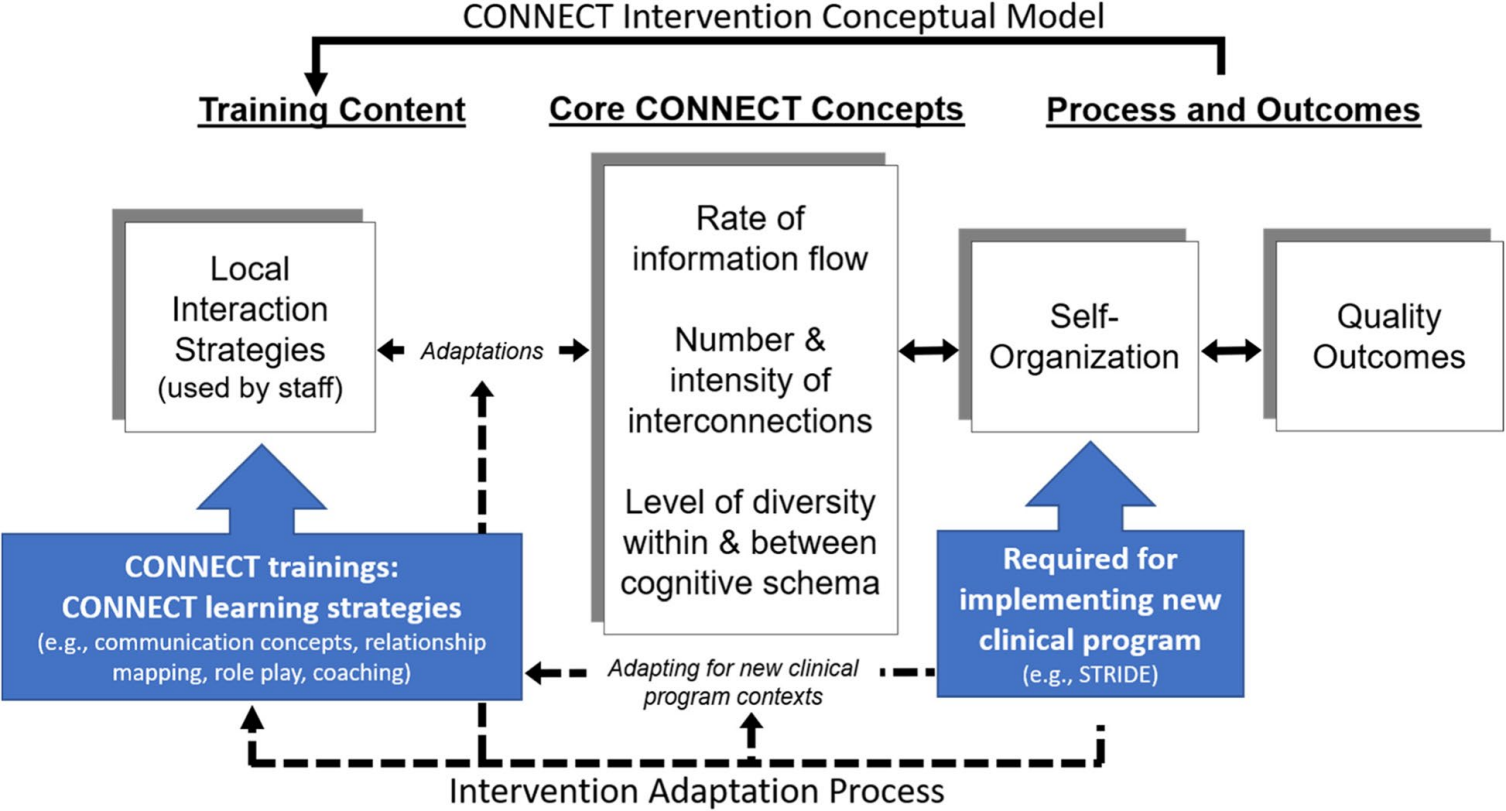
Conceptualizing intervention adaptations to new program context: The *CONNECT for Quality* intervention

In preparation for modifying *CONNECT for Quality* to *CONNECT for STRIDE*, adaptations were informed by a combination of the Planned Adaptation Framework [6] and the Framework for Reporting Adaptations and Modifications (FRAME) [9, 13]. Although both frameworks offer systematic approaches to modifying interventions, they reflect change at different phases of the intervention refinement process. Use of a single approach may yield limited detail describing and justifying changes. To lay the groundwork for modifications of *CONNECT for STRIDE* content and delivery format, we followed the Planned Adaptation Framework [6] to consider the clinical and contextual differences between applications of *CONNECT for Quality* in its original and new setting (Fig. 1). Next, we worked with interventionists from *CONNECT for Quality* to explore clinical and contextual differences between *CONNECT for Quality’s* original and new settings and to identify core components of *CONNECT for Quality* for improving team interactions and information exchange. Non-core elements of programmatic structure, content, and language were tailored for hospital care settings based on considerations of contexts of clinical service delivery. To maximize fidelity with intervention content, we worked closely with *CONNECT for Quality* investigators and interventionists for planned modifications to content, specifically for STRIDE implementation. Adaptations to *CONNECT for Quality* were first proposed by interventionists who reviewed the original *CONNECT for Quality* intervention’s peer-reviewed literature (conceptual development, protocol, and evaluations), *CONNECT for Quality* documents and training materials to identify core and non-core elements of *CONNECT for Quality*. These elements, contextual differences between nursing home-facing and hospital ward-facing *CONNECT for Quality*, and proposed adaptations were verified and confirmed with the developers of the original *CONNECT for Quality* intervention. We used the expanded FRAME for reporting intervention modifications, documenting both planned and unplanned modifications that occurred throughout refinement of *CONNECT for STRIDE* and its deployment, which can later be explicitly linked to outcomes for evaluation.

Observational data were collected during delivery of *CONNECT for STRIDE* to sites implementing the STRIDE program. Information regarding participation and reach of CONNECT activities were obtained through invitation and participant attendance logs of in-person *CONNECT for STRIDE* sessions at hospital sites as well as participation and engagement in post-*CONNECT for STRIDE* training follow-up activities (described in further detail below). Assessment of *CONNECT for STRIDE* participation was based on rates of attendance of at least 1 session of *CONNECT for STRIDE* on-site training activities among associated hospital units and staff invited by each hospital’s STRIDE implementation leadership team. This was ascertained at two levels, a) individual staff participation and b) roles/departments represented. As part of the formal program evaluation of STRIDE program implementation processes and effectiveness (forthcoming), we conducted one-on-one interviews by telephone or in-person with STRIDE delivery team members who participated in *CONNECT for STRIDE* trainings (*n* = 23) to assess satisfaction and perceived effectiveness of CONNECT. Using directed content analysis [25], qualitative analysts summarized responses to questions about *CONNECT for STRIDE* by site and a priori domain (e.g., how *CONNECT for STRIDE* guided changes in work; how it changed staff communications; which and how *CONNECT for STRIDE* strategies were used to improve work) in a framework matrix to identify patterns in reported experience. For this paper, we describe initial experiences with *CONNECT for STRIDE* across sites, which reflects the feasibility and acceptability of our modifications to the original *CONNECT for Quality* intervention. Staff qualitative interviews were approved as research by the Institutional Review Board of the Durham VA HCS.

## Results

In this section, we use the combined Planned Adaptations and FRAME frameworks to report the changes to the *CONNECT for Quality* intervention for *CONNECT for STRIDE*. This includes planned and unplanned adaptations and modifications to its core components (*CONNECT for Quality* concepts and training content, Table 2) and non-core components (delivery of *CONNECT for STRIDE*, Table 3).

**Table 2:**
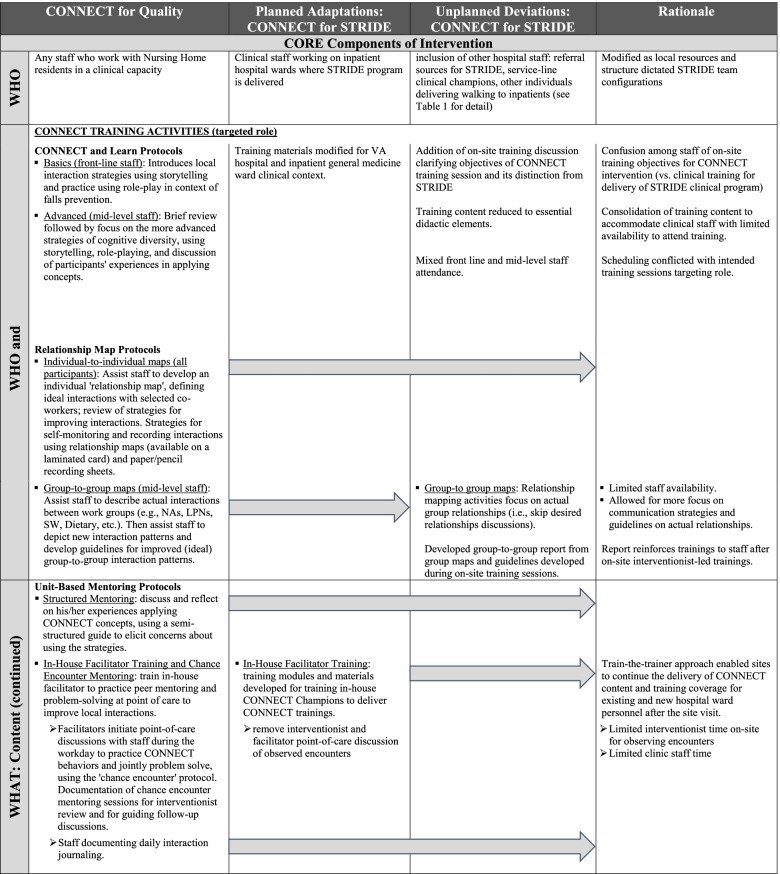
Description of modifications of CONNECT interventions’ core components: Intervention Context [6, 9, 13]

**Table 3:**
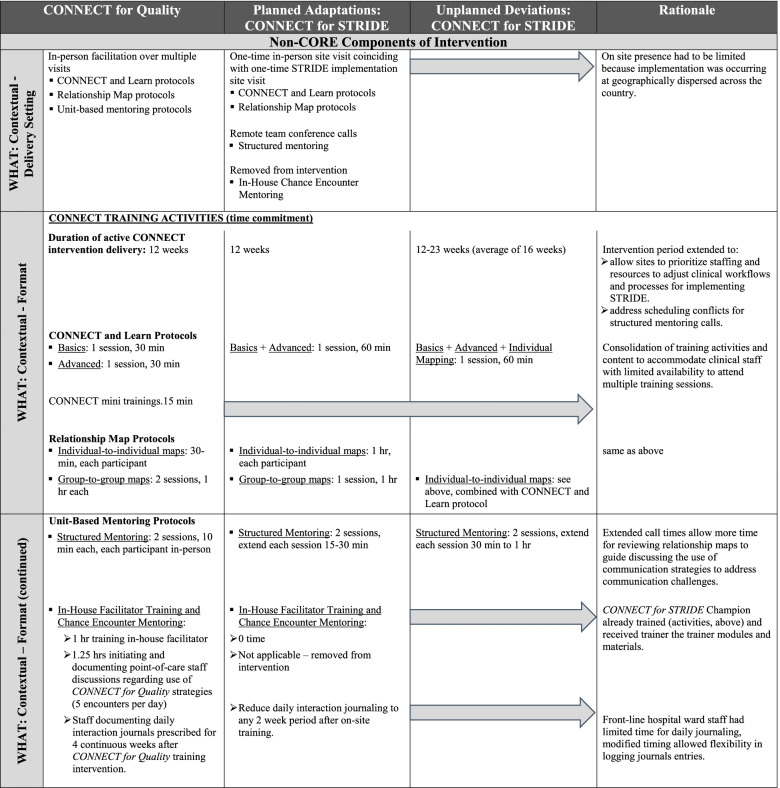
Description of modifications of CONNECT interventions’ non-core components: Intervention delivery [6, 9, 13]

### Planned adaptations

#### Identifying core components of *CONNECT for STRIDE* for improving team interactions and information exchange: training concepts and content

The Planned Adaptation Framework was also used to identify core and non-core components of *CONNECT for Quality* as originally designed, before applying changes to the intervention design. These changes and the process of documenting them were guided by Stirman and colleagues’ FRAME [9, 13].

Planned modifications of the original *CONNECT for Quality* intervention first considered core elements of the intervention and preserved these “active ingredients” in its application to STRIDE implementation [6]. The core components of *CONNECT for Quality* intervention are the training concepts and content based on complexity science and social learning theories [15–17]. Mechanisms of social learning – communication and information flow, interactions, and relationship networks [26] – are reflected in *CONNECT for Quality* training content, designed to assist clinical staff in evaluating their relationships with their coworkers (relationship mapping), sensemaking from multiple perspectives, practicing communication strategies to improve connection, and setting goals for improving communication and relationships.

*CONNECT for Quality* training content is organized into a series of activities (Table 2, Column 1). To facilitate interdisciplinary learning and create new connections among nursing home staff, in-person training sessions (“CONNECT and Learn”) comprised of a series of didactic learning sessions with intervention participants, introduced concepts in communication and cognitive diversity and practical application of communication strategies through story-telling and interactive role play. To define and evaluate improvement in interactions, relationship mapping activities involved didactic learning and guided exercises evaluating individual- and group-level relationships in participants’ care teams. Using the core principles introduced in the CONNECT and Learn sessions, participants then developed goals and guidelines for improving communication and interaction in these relationships. In-house facilitators were trained to practice peer mentoring and problem-solving point of care discussions to improve local interactions. At the end of formal, in-person training activities, *CONNECT for STRDE* interventionists provided materials (e.g., training workbooks, bookmarks with printed communication and interaction strategies, and group-to-group interaction mappings for display in staff workrooms) to reinforce and encourage the use of communication tools and strategies.

After *CONNECT for STRIDE* interventionist-led trainings were completed, participants were asked to self-monitor and journal communication patterns and the use of interaction strategies introduced in CONNECT and Learn sessions. This follow-up activity enabled participants to directly and independently apply *CONNECT for STRIDE* principles and mappings to interactions in their care teams. Clinical staff recorded daily interactions and forwarded them to interventionists, to inform their structured mentoring follow-up sessions with individual staff to assess progress and problem solve barriers.

There were minimal modifications to the content of *CONNECT for STRIDE*. Didactic trainings maintained the same objectives, communication concepts and activities because they are core components of the underlying complexity science theory from which *CONNECT for Quality* is based [16]. Since the trainings were based on constructivist learning principles [15, 17] and found to be acceptable to a range of adult learners and found to be acceptable to a range of adult learners [14, 20], didactic in-person training (e.g., CONNECT and Learn, guided relationship mapping activities, and structured mentoring activities) was preserved in *CONNECT for STRIDE* (Table 2). However, group-based activities’ vignettes and role-play case scenarios were modified for the new target audience and clinical context for *CONNECT for STRIDE* (i.e., care on inpatient wards). In addition, due to the limited presence of research interventionists and clinic staff time, we removed *CONNECT for STRIDE* interventionist and in-house facilitators’ point of care coaching of observed encounters. As a result, the in-house facilitator training materials were modified for training in-house *CONNECT for STRIDE* Champions who would train new staff (i.e., train the trainer approach, detailed further below).

#### Identifying non-core components of *CONNECT for STRIDE*: Planned modifications for intervention delivery in new clinical context

Much of the planned adaptations to *CONNECT for Quality* centered on the non-core elements necessary for tailoring to STRIDE implementation for inpatient hospital teams (Table 3). Notably, the clinical context of falls prevention in *CONNECT for Quality* differed from inpatient mobility during acute hospital stays in *CONNECT for STRIDE*, which not only required different personnel to be exposed to the intervention. As a result, modifications were also made to features of delivering *CONNECT for STRIDE*, in terms of its programmatic structure and modality of delivery, as well as format. As described in Table 3 Column 1, activities in the original *CONNECT for Quality* intervention occurred in nursing homes over 12-weeks, with interventionists appearing on-site for a total of five in-person didactic trainings convening over several weeks in sessions ranging from 30 to 70 min each. An abridged version of *CONNECT for STRIDE* didactic materials was developed for “CONNECT Mini” trainings that were delivered to hospital staff with scheduling conflicts unable to attend the research team’s on-site sessions or shared with in-house *CONNECT for STRIDE* Champions for new staff onboarding. Follow-up facilitation and mentoring activities occurred separately and were scheduled in two 10-min increments for up to 70 min (cumulatively) with individual clinic staff.

*CONNECT for STRIDE* activities retained the 12-week intervention design, but the timeline for implementing the STRIDE program and expanded geography of participating inpatient hospital wards across multiple VA medical centers made it impractical to replicate the *CONNECT for Quality* in-person activity schedule for STRIDE implementation. Rather, *CONNECT for STRIDE* didactic training sessions and guided exercises continued to be conducted in-person and on-site to coincide with a one-time STRIDE implementation site visit prior to hospitals’ rollout of STRIDE training and delivery (Table 3, Column 2). *CONNECT for STRIDE* activities were also consolidated and reorganized – training sessions were shortened by combining content to accommodate the schedules of hospital staff (particularly nurses, CNAs, and physical therapists responsible for direct patient care). As a result, didactic sessions on CONNECT and Learn and the guided interaction mapping activities were presented in a single, consolidated day of training at hospitals (5 sessions in *CONNECT for Quality* vs. 3 sessions in *CONNECT for STRIDE*). Follow-up activities with STRIDE personnel were also modified in terms of frequency and delivery modality. While less frequent and occurring remotely, this modified schedule of follow-up activities enabled reflection, coaching/facilitation, and reinforcement of communication concepts and strategies introduced during the initial on-site trainings.

To reinforce and support local use of the communication tools and strategies from *CONNECT for STRIDE*, interventionists worked with site-level STRIDE implementation leadership to identify and train existing personnel to serve as internal facilitators (i.e., *CONNECT for STRIDE* Champions). This train-the-trainer approach enabled sites to continue the delivery of *CONNECT for STRIDE* content and training coverage for existing and new hospital ward personnel and encourage the use of concepts and tools among hospital ward staff. By assisting the collection of staff-reported interaction documents, *CONNECT for STRIDE* Champions also served as liaisons between *CONNECT for STRIDE* participants at sites and research interventionists.

### Unplanned modifications to *CONNECT for STRIDE*

Initial experiences delivering *CONNECT for STRIDE* at hospitals were unpredictable and logistical adjustments made with sites led to additional unplanned refinements and modifications of the delivery that were also documented according to the FRAME [9, 13]. First, hospitals implementing the STRIDE program began defining their teams in various configurations for delivering the STRIDE program, as local resources and structure dictated, to also include referral sources to the STRIDE program, and clinical champions across service lines. As a result, the intended audience for *CONNECT for STRIDE* expanded (Table 1). On-site training sessions were intended to be conducted separately for frontline staff and mid-level program managers (e.g., individual mapping for front line staff and group-to-group mapping for mid-level staff), but challenges of coordinating separate trainings with coverage for inpatient care resulted in mixed group attendance.

Next, despite a priori planned adaptations to *CONNECT for STRIDE* program structure and delivery, it was further modified to better align with the clinical practice for STRIDE implementation, for which *CONNECT for STRIDE* activities were designed to support (Table 3, Column 3). For example, although it was our intent to deliver *CONNECT for STRIDE* in the same 12-week intervention timeline as the original *CONNECT for Quality*, we expanded the *CONNECT for STRIDE* timeline from 12 to 23 weeks at hospitals (an average of 16 weeks) to accommodate sites needing prioritize their staffing and resources to adjust clinical workflows and processes for implementing the STRIDE program. In addition, when sites were not able to release staff from clinical duties for more than one hour, CONNECT and Learn and Individual Mapping training sessions were further consolidated into two sessions. Follow-up structured mentoring calls with *CONNECT for STRIDE* participants were further extended, in order to allow sufficient time for interventionists to remotely review relationship maps and guide substantive follow-up discussion about the use of communication strategies to improve challenges in clinical team interactions.

During the one-time STRIDE implementation site visits (by STRIDE program implementation specialists and *CONNECT for STRIDE* interventionists) at the participating sites receiving *CONNECT for STRIDE*, additional modifications were made to content to address confusion around the purpose of *CONNECT for STRIDE* staff trainings versus clinical trainings for delivery of STRIDE (Table 2, Column 3). Due to scheduling constraints of hospital staff, didactic training and guided relationship mapping activities were further pared down to essential didactic elements. This provided more time for meaningful discussion and development of communication strategies to improve the strength and quality of group interactions and information exchange.

### Preliminary experiences

We describe CONNECT for STRIDE participants’ initial experiences to determine preliminary feasibility and acceptability of *CONNECT for STRIDE* modifications for STRIDE implementation. Overall *CONNECT for STRIDE* was delivered to one-third of invited clinicians and staff, though participation rates varied widely across sites (range of participation = 14 to 81%; Table 4). All disciplines and personnel roles on hospital STRIDE teams participated in *CONNECT for STRIDE* intervention on-site trainings. Engagement in *CONNECT for STRIDE* activities diminished when the intervention moved to remote, phone-based intervention follow-up activities after the in-person trainings at site visits (versus in-person follow-up sessions in the original *CONNECT for Quality* intervention). As noted above, unplanned adaptations to the intervention occurred before and during delivery at the four participating sites randomized to *CONNECT for STRIDE*. These modifications during *CONNECT for STRIDE* delivery did not appear to affect participation. Instead, the variation in participation may be due to factors specific to hospitals (e.g., inpatient and staffing capacity).

**Table 4:** CONNECT for STRIDE participation at hospitals implementing new STRIDE program

	**Site A**	**Site B**	**Site C**	**Site D**
***CONNECT for STRIDE on-site training sessions***				
Individual participation^a^: Hospital STRIDE members				
Invited to intervention training sessions, Overall N	168	66	47	146
Participated in training sessions, Overall N	23	18	38	66
Participation Rate, Overall %	14%	27%	81%	45%
Frontline personnel, N (%)^b^	17 (12%)	12 (25%)	30 (77%)	57 (42%)
Mid-level personnel, N (%)^b^	6 (19%)	6 (33%)	8 (100%)	9 (90%)
Group-level participation^a^: Hospital STRIDE team roles and service lines				
Role types in training sessions, N (penetration %)	9 (100%)	6 (54%)	8 (100%)	9 (150%)
Service lines in training sessions, N (penetration %)	3 (100%)	4 (100%)	3 (100%)	4 (100%)
***CONNECT for STRIDE Training: Individual Follow-up Activities***				
Participated in CONNECT *for STRIDE* Individual Mapping Session, N	15	15	15	45
Completed ≥ 1 self-monitored interaction report, N (%)	12 (80%)	9 (60%)	7 (47%)	5 (11%)
Participated in *CONNECT for STRIDE* Group to Group Mapping Session, N	6	6	6	8
Completed ≥ 1 structured mentoring call with CONNECT *for STRIDE* interventionist, N (%)	4 (66%)	3 (50%)	5 (83%)	4 (50%)
Completed all planned structured mentoring calls with *CONNECT for STRIDE* interventionist, N (%)	2 (33%)	3 (50%)	1 (16%)	2 (25%)

^a^Participation defined as attendance in ≥ 1 training session

^b^Denominators for level-specific personnel participation rates are the number of frontline and mid-level personnel roles invited to participate in *CONNECT for STRIDE* trainings (not reported)

From our qualitative interviews with staff across sites, respondents had mixed views about acceptability of *CONNECT for STRIDE*, ranging from positive to neutral. Some respondents reported that *CONNECT for STRIDE* provided status checks on the strength and appropriateness of relationships between staff, highlighting areas for improvement across hospital teams’ groups.

*CONNECT* (for *STRIDE*)* showed us that we had a kind of strong and appropriate relationship between nursing and therapy staff…maybe a little bit weaker relationship with leadership*.

Moreover, others reporting increased willingness to communicate views and awareness of the perspectives of other staff. This in turn enhanced teamwork and work culture on the hospital wards.

*A lot of people walked out of the training saying that was really helpful. We all could benefit from having that intra-perspective of how we relate to others. And I know a lot of people kind of changed their attitudes after going to those trainings.*

*…the certified nursing assistants will talk to the doctors more, for example, than maybe they did before. Because they feel like they have important information to impact. So it’s just little things that make such a huge difference in the culture.*

However, other *CONNECT for STRIDE* participants did not perceive any impact for staff-level interactions on the general medicine hospital ward. One participant reported that.

*honestly I did not find it super useful… I think … they wanted the chief of staff in that, you had some high-level leaders and just mapping everything out to that level of detail was, I just, it got tedious. Let’s just say it got tedious. And it did not see very much value in that.*

Another participant reported that members involved in delivering the STRIDE program already and frequently communicated amongst themselves [physical therapists] and with the nurses on the unit, noting that “I don’t think it [*CONNECT for STRIDE*] changed anything significant in terms of how we communicate”.

## Discussion

To disseminate clinical interventions for expanded implementation, it is critical to consider modifications for successful application in new settings. In this paper, we describe the process of adapting a provider facing, team-based intervention for use in a new, hospital-based clinical context. The limited detail in intervention adaptations noted by Copeland and colleagues not only hinders rigorous evaluation of effectiveness of interventions and their adaptations [11], but may also signal the shortcomings of approaches to intervention modifications. In the case study presented here, we found incorporating approaches from the Planned Adaptation Framework and Framework for Reporting Adaptations and Modifications was useful for organizing the modifications in a systematic way. Single, standalone use of either approach would not provide adequate methodologic detail and justification of changes. First, Lee and colleagues’ Planned Adaptation Framework was useful for systematically planning modifications to interventions, by identifying key contextual differences in the contexts of intervention delivery, intervention mechanisms of change, and core and non-core elements of intervention design. Even with planning, the unpredictability of implementing pragmatic interventions in real-world settings commonly prompts unplanned modifications. Stirman Wiltsey and colleagues’ FRAME provided a structure for documenting both planned and unplanned intervention adaptations. Thus, the Planned Adaptation Framework and FRAME are good complements that, when combined, comprehensively specify core vs. non-core elements, rationale, and applied changes to interventions to be scaled for other topical and organizational contexts.

A key advantage of this hybrid approach is comprehensive, a priori planning of anticipated changes and recording of decisions and rationale of executed changes, from original design to expected and unexpected changes over time. This not only provides rich detail underlying intervention modifications, informing continuous, real-time assessment of impacts of adaptations in the field but ultimately enables evaluation of intervention effectiveness or comparative evaluation of the intervention across clinical contexts and settings. Future research will empirically assess the effectiveness of this initial experience adapting *CONNECT for STRIDE* for healthcare teams in hospital settings and also inform considerations for further modifications to optimize provider experience and clinical program outcomes for scale up implementation of the STRIDE program and the use of *CONNECT for Quality* in other program implementation contexts.

## Conclusions

Comprehensive documentation on intervention design modifications is essential to enabling ongoing intervention development and refinement in new settings. Our hybrid planning and documentation approach, using the Planned Adaptation Framework and FRAME, to applying modifications to new application of a provider-facing clinical intervention serves a useful case study for guiding similar intervention adaptations.

## Data Availability

The U.S. Department of Veterans Affairs (VA) places legal restrictions on access to VA data, which includes both identifying data and sensitive information. The analytic data sets used for this study are not permitted to leave the VA firewall without a Data Use Agreement, consistent with other studies based on VA data.

## Abbreviations

CNA: Certified Nurse Assistant
FRAME: Framework for Reporting Adaptations and Modifications-Enhanced
Function QUERI: Optimizing Function and Independence Quality Enhancement Research Initiative
MD: Medical Doctor
NP: Nurse Practitioner
PA: Physician Assistant
STRIDE: assiSTed eaRly mobIlity for hospitalizeD older vEterans
VA: Veterans Affairs
